# Palliative care for patients with glioma: A recent scientometric analysis of the Web of Science in 2022

**DOI:** 10.3389/fonc.2022.995639

**Published:** 2022-12-13

**Authors:** Zhiyuan Xiao, Wenlin Chen, Haiyan Zhao, Hai Wang, Binghao Zhao, Delin Liu, Tianrui Yang, Tingyu Liang, Hao Xing, Yaning Wang, Yuekun Wang, Xiaopeng Guo, Yi Zhang, Yu Wang, Wenbin Ma

**Affiliations:** Department of Neurosurgery, Peking Union Medical College Hospital, Chinese Academy of Medical Sciences and Peking Union Medical College, Beijing, China

**Keywords:** citation classics, citations, palliative care, hospice care, glioma, scientometric analysis

## Abstract

**Background:**

Patients with glioma present with complex palliative care needs throughout their disease trajectory. A scientometric analysis is effective and widely used to summarize the most influential studies within a certain field. We present the first scientometric analysis of palliative care for patients with glioma.

**Methods:**

Based on a Preferred Reporting Items for Systematic reviews and Meta-Analyses (PRISMA) principle, we conducted a generalized search for articles on palliative care for glioma in the Web of Science database and evaluated the top 100 most frequently cited articles among 2,542 articles.

**Results:**

The number of citations for the top 100 cited articles on palliative care for glioma ranged from 10 to 223. We have a narrative conclusion, as follows: most of these articles were published in oncology-specific journals (n = 53) and palliative-specific journals (n = 22). The United States, Australia, and the Netherlands were the top three countries contributing most of the articles (n = 59). Most of the research methods were quantitative analyses, qualitative analyses, and systematic reviews and meta-analyses (n = 70). In quantitative studies, 66 scales were used, and the top three scales used included the following: the Distress Thermometer, Functional Assessment of Cancer Therapy-Brain Index (FACT-Br), and Karnofsky Performance Scale (KPS). The articles were classified into six major categories based on research subjects, including patients (n = 44), caregivers (n = 16), patients and caregivers (n = 20), literature (n = 19), and healthcare providers (n = 1). Articles were classified into seven major categories based on research themes: quality of life (n = 11); end-of-life symptoms and care (n = 16); palliative and supportive care needs (n = 35); advance care planning and decision making (n = 4); psychological, social, and spiritual needs (n = 12); hospice utilization and referral (n = 3); and others (n = 19). The studies of the primary topic are correlated with the number of citations.

**Conclusions:**

The results of the analysis indicated that patients diagnosed with glioma present a high variety of palliative care needs, including physical, psychological, social, and spiritual needs. The caregiver’s burden and needs are important as well. The proportion of quantitative analyses, qualitative analyses, and systematic reviews and meta-analyses is relatively high, but the number of randomized controlled trials (RCTs) was low. End-of-life care and supportive care needs appeared frequently. Thus, palliative care is an urgent need to be addressed in glioma management. The appropriate scales should be selected for patients with glioma and meet their palliative needs.

## Introduction

Gliomas are the most frequent primary tumors of the central nervous system, and the 2021 WHO classification of tumors of the central nervous system combined histological features and molecular markers to improve the classification, diagnostic criteria, and grading of gliomas (Grading of adult diffuse gliomas according to 2021 WHO Classification of Tumors of the Central Nervous System) (1–4). While the effective treatment of glioma is limited, and the clinical treatment of glioma includes surgery, radiotherapy, chemotherapy, targeted therapy, immunotherapy, and novel therapies (5–10). Although comprehensive treatments are used, glioma patients have poor prognoses, especially for glioblastoma (GBM). The recurrence rate is high, the reported 5-year survival rate of patients with GBM remains less than 10%, and the median survival is still less than 2 years. Patients who underwent gross total resection had a median overall survival (OS) of 14.53 months, while patients who underwent subtotal resection had a median survival (OS) of 10.44 months (11). General and disease-specific symptoms are common in the disease trajectory, especially in the end-of-life phase. All of these symptoms result in difficult and complex situations for the patient. Family caregivers face a high level of distress as well. In this complex situation, palliative and supportive care become more and more important, and the demand increases.

Modern hospice and palliative care began at St. Christopher’s Hospice in London, England, and continues to expand today (12–16). The definition of palliative care provided by the WHO is an approach that improves the quality of life of patients and their families facing the problems associated with life-threatening illness through the prevention and relief of suffering based on the early identification and impeccable assessment and treatment of pain and other problems, including physical, psychosocial, and spiritual needs (17). An update indicated that palliative care is a crucial part of integrated, people-centered health services. Relieving serious health-related suffering, regardless of whether it is physical, psychological, social, or spiritual suffering, is a global ethical responsibility (18). International Association for Hospice and Palliative Care (IAHPC), as one of the partners of WHO, has its origins in the International Hospice Institute, founded by Josefina Magno, MD, in 1980 (19). IAHPC is a global non-profit membership organization whose vision is a world free from health-related suffering with universal access to quality palliative care. The definition of palliative care provided by IAHPC is the active holistic care of individuals across all ages with serious health-related suffering due to severe illness, particularly the suffering of those near the end of life. Palliative care aims to improve the quality of life of not only patients but also their families and their caregivers (20). Due to the limitations associated with the nature of the disease, high recurrence rate, and short overall survival of patients with high-grade glioma (WHO 3–4), patients experience a complex condition during the disease trajectory. Patients experience the following common progressive focal neurological deficits with a wide range of symptoms at the end of life: cognitive deficits, paralysis, seizures, fatigue, dysphagia, headaches, drowsiness, loss of consciousness, incontinence, and psychosocial burden (21–23). Patients with high-grade glioma have higher demands for palliative care.

Scientometrics is a discipline that quantitatively analyzes information from the literature mainly using mathematical and statistical methods. The number of citations of an article may reflect the degree of concern or value of the topic in question to a certain extent, as well as the importance, influence, and quality of the article. Scientometric analysis methods have been applied to investigate different cancers treated using neurosurgery (24–26), but not to palliative care for glioma. In this study, we mainly used a scientometric method to statistically analyze the most frequently cited literature on palliative care for glioma and to summarize current research and the prospects of palliative care for patients with glioma. This article mainly analyzed the top-cited studies on palliative care for glioma, and current research hotspots and possible research directions were derived based on the results. By referring to this article, readers can quickly comprehend the major research topics on palliative care for glioma and where to find them, which will guide the direction of future studies and publications.

## Materials and methods

### Search strategy and selection criteria

A search of the Web of Science database was conducted on 7 April 2022, using topics and all field strategies with the following terms: (“palliative care” or “palliative medicine” or “Hospice and palliative care nursing” or “Terminal care” or “Hospice care” or “Advance care planning” or “Early palliative” or “Decision-making in the end of life” or “Quality of life in the end of life” or “limitation of life support” or “symptom burden” or “caregiver*” or “physical need* “ or “psychological need*” or “psychosocial need*” or “spiritual need*” or “social need*”) and (“High-grade glioma*” or “Glioblastoma*” or “HGG” or “intracranial glioma*” or “glioma*” or “malignant glioma*” or “malignant cerebral glioma*” or “primary malignant brain tumo*” or “primary malignant brain neoplasm*” or “malignant primary brain tumo*” or “malignant primary brain neoplasm*” or “GBM” or “glioblastoma multiforme” or “Neuro-oncologist*” or “Neuro-oncology” or “Neurosurgery or Neuro-oncological”). No restrictions were placed on the publication date, language, document types, or Web of Science categories. The articles were subjected to two rounds of selection: published articles were first screened by reading the title for their relevance to palliative care and glioma, and the abstracts of the remaining articles were reviewed. Articles not directly pertaining to palliative care and glioma were excluded. A total of 130 publications were included in descending order. The Pareto principle (27), which holds that a small number of factors have a disproportionate impact on any outcome, was used in 130 publications, and we identified the top 100 most-cited articles published between 2003 and 2020 on palliative care for patients with glioma that may be considered significant and impactful works, as well as the most noteworthy. However, 30 lowest-cited publications with less than the number of 10 citations were not included in this study.

### Preferred reporting items for systematic reviews and meta-analyses criteria

The Preferred Reporting Items for Systematic reviews and Meta-Analyses (PRISMA) statement consists of a 27-item checklist and a four-phase flow diagram that was published in 2009 (28) and has been updated to PRISMA 2020 version. It was designed to help authors prepare transparent accounts of their reviews, and its recommendations have been widely endorsed and adopted (29). The PRISMA flow diagram depicts the flow of information through the different phases of a systematic review. It maps out the number of records identified and included and the reasons for the exclusion (30, 31). We completed the identification of the studies under the PRISMA criteria.

### Data collection

The following parameters were extracted from each article: title, first author, corresponding author, country, institution, journal, impact factor (IF) of the journal, category of the journal, total citation count, annual citation count, and publication year. The country of origin was based on the affiliation of the corresponding author. Articles were classified according to the study design and topical theme. Article types consisted of quantitative analyses, qualitative analyses, systematic reviews and meta-analyses, mixed-methods research, cohort studies, cross-sectional studies, case reviews, guidelines, and randomized clinical trials (RCTs). Articles were classified based on their primary theme to assess trends in the literature. These themes were chosen to represent major areas of palliative care in glioma research and encompass important aspects relevant to clinical practice. The themes included quality of life, end-of-life symptoms and care, palliative and supportive care needs, advance care planning and decision-making, psychosocial and spiritual needs, and hospice utilization and referral. A pie chart was drawn to describe the national contribution, and a line chart was drawn to describe the relationship between the number of published papers and research topics, subjects, and methods.

### Statistics

Correlation analyses of continuous variables were conducted using SPSS version 26 (IBM Corp., Armonk, NY, USA) and GraphPad Prism 9 (GraphPad, La Jolla, CA, USA). Continuous data were expressed as mean ± standard deviation or median for normally or non-normally distributed data, respectively. The normality of the data was analyzed by the one-sample Kolmogorov–Smirnov test. The differences between groups with the t-test were tested when data were normally distributed and the variance was homogeneous. The Mann–Whitney U test was used when data were skewed. Spearman’s correlation analysis was used for the non-normality of the data, and a two-sided p-value <0.05 was considered statistically significant.

### Financial and material support

This work was supported by grants from the National Natural Science Foundation of China (grant number 82151302), the Beijing Municipal Natural Science Foundation (grant number 7202150), the Beijing Municipal Natural Science Foundation [grant number 19JCZDJC64200(Z)], and the Tsinghua University-Peking Union Medical College Hospital Initiative Scientific Research Program (grant number 2019ZLH101).

## Results

**Figure 1:**
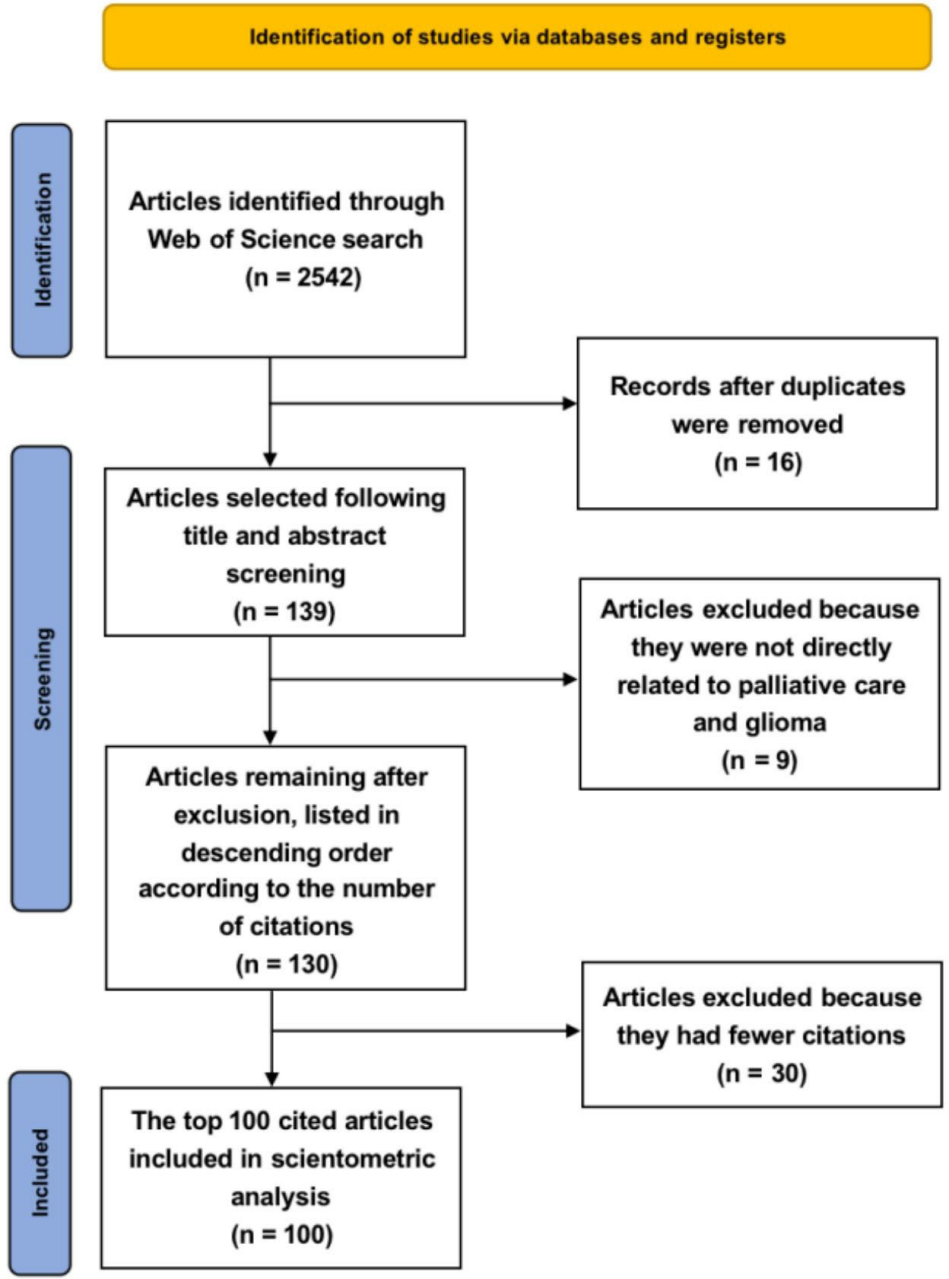
Flowchart of the literature screening process in the PRISMA style format (28–31). PRISMA, Preferred Reporting Items for Systematic reviews and Meta-Analyses.

Among the final list of studies obtained, the top 100 cited articles following the PRISMA principle were included in the final analysis (**Figure 1**). Our search query yielded 2,542 articles, with a total of 3,809 citations. The top 100 cited articles are listed in **Supplementary Table 1**. Among the top-cited articles, the most-cited article was referenced 223 times, and the least-cited article was referenced 10 times. The mean and standard deviation of all articles’ total citation counts was 38.09 ± 32.77 (median, 27), and the mean and standard deviation of IFs were 6.59 ± 25.38 (median, 3.6). The details of the top-cited articles were analyzed.

An assessment of the sources of the articles showed that most of the top-cited articles were published in oncology-specific journals (n = 53) or palliative-specific journals (n = 22). Among the remaining articles, 25 were published in journals from various other categories. *Neuro-Oncology* and the *Journal of Neuro-Oncology* published the most top-cited articles (35 articles), and *Supportive Care in Cancer* published 17 articles (**Table 1**). The IFs of the journals and the number of citations showed non-normality distributions (Spearman’s correlation coefficient = 0.142; p > 0.05). The IFs of the journals are not correlated with the number of citations. When the journals were divided into two groups, the open-access journals and the standard published journals, there was no statistical difference in the number of citations (p = 0.35; p > 0.05) and the IFs of journals (p = 0.215; p > 0.05).

**Table 1:** Journal rankings based on publications of the top 100 cited articles on palliative care for patients with glioma.

Journal	Journal category	Publication numbers	Impact factor
*Journal of Neuro-Oncology*	Oncology; clinical neurology	24	3.639
*Supportive Care in Cancer*	Oncology; healthcare sciences and services; rehabilitation	17	2.967
*Neuro-Oncology*	Oncology; clinical neurology	11	7.137
*Patient Education and Counseling*	Public, environmental, and occupational health; social sciences, interdisciplinary	4	2.555
*Frontiers in Oncology*	Oncology	4	6.244
*European Journal of Oncology Nursing*	Oncology; nursing	3	2.116
*Psycho-Oncology*	Oncology; psychology; psychology, multidisciplinary; social sciences, biomedical	3	3.011
*European Journal of Cancer Care*	Oncology; healthcare sciences and services; nursing; rehabilitation	3	2.183
*Palliative Medicine*	Healthcare sciences and services; public, environmental and occupational health; medicine, general and internal	2	3.973
*Oncology Nursing Forum*	Oncology; nursing	2	1.352
*Journal of Neuroscience Nursing*	Clinical neurology; nursing	2	0.906
*Cancer*	Oncology	2	6.86
*European Journal of Cancer*	Oncology	1	6.512
*PLOS One*	Multidisciplinary sciences	1	3.041
*Oncologist*	Oncology	1	4.33
*Neurosurgical Focus*	Clinical neurology; surgery	1	2.857
*Neuropsychological Rehabilitation*	Neurosciences; psychology	1	2.503
*Medicine*	Medicine, general and internal	1	1.644
*Lancet Oncology*	Oncology	1	31.003
*Journal of Palliative Medicine*	Healthcare sciences and services	1	1.652
*Journal of Clinical Oncology*	Oncology	1	12.287
*Journal of Clinical Nursing*	Nursing	1	2.767
*Journal of Cancer Survivorship*	Oncology; social sciences, biomedical	1	3.671
*Health and Quality of Life Outcomes*	Healthcare sciences and services; health policy and services	1	2.965
*Disability and Rehabilitation*	Rehabilitation	1	2.16
*Current Opinion in Oncology*	Oncology	1	2.955
*Current Oncology*	Oncology	1	2.936
*Clinical Neurology and Neurosurgery*	Clinical neurology; surgery	1	1.78
*Cancers*	Oncology	1	6.012
*Cancer Nursing*	Oncology; nursing	1	2.03
*Canadian Medical Association Journal*	Medicine, general and internal	1	2.485
*Ca-A Cancer Journal for Clinicians*	Oncology	1	255.732
*Brain Sciences*	Neurosciences	1	3.114
*BMJ Supportive & Palliative Care*	Healthcare sciences and services	1	3.568
*American Journal of Hospice & Palliative Medicine*	Healthcare sciences and services	1	1.808

**Figure 2:**
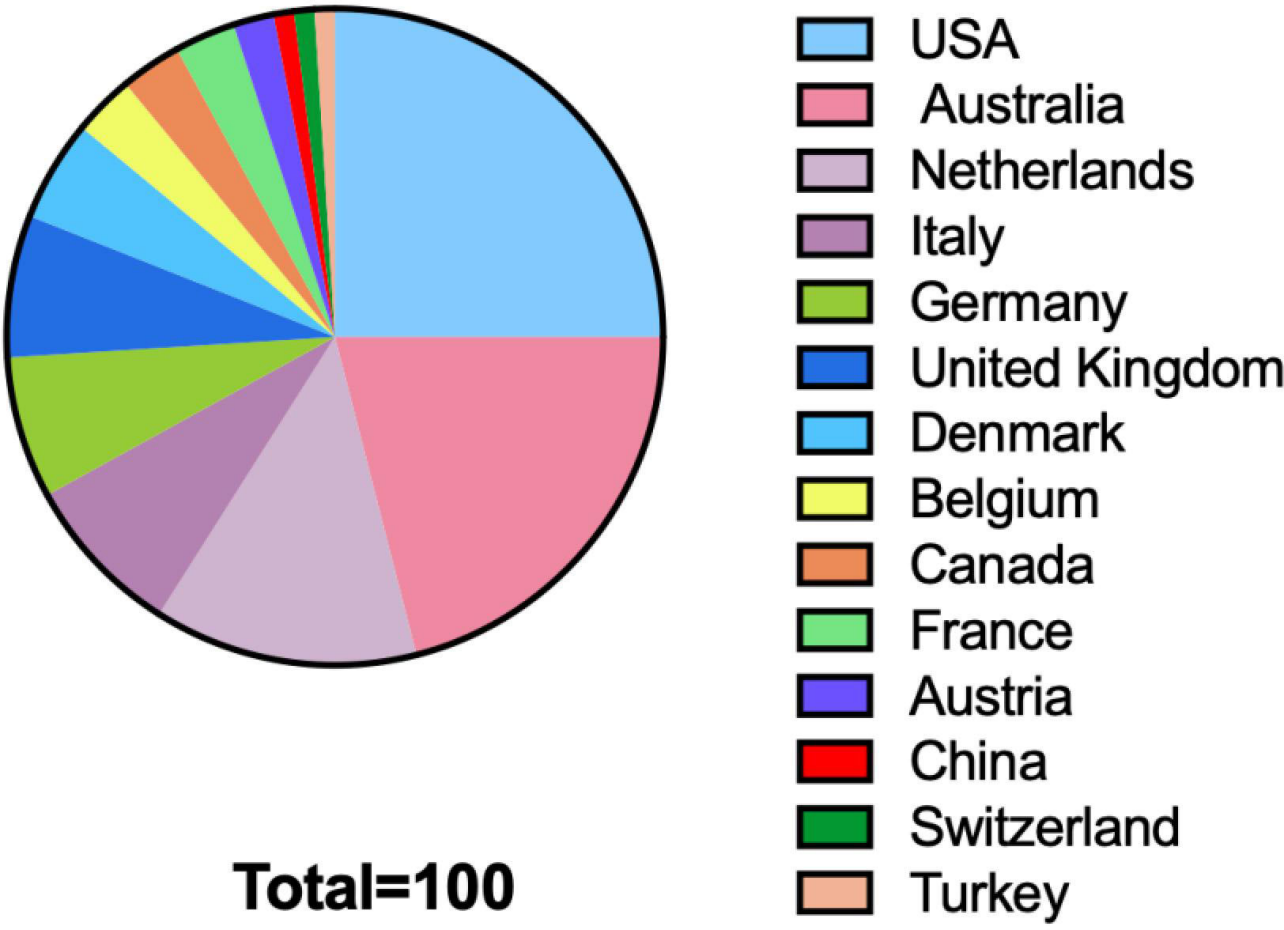
Countries contributing to the top 100 cited articles on palliative care issues of patients with glioma.

Overall, 14 countries contributed to the top 100 cited articles (**Figure 2**), with institutions from the United States contributing the greatest number (25 articles), followed by Australia (21 articles), the Netherlands (13 articles), Italy (8 articles), Germany (7 articles), the United Kingdom (7 articles), Denmark (5 articles), Belgium (3 articles), Canada (3 articles), France (3 articles), Austria (2 articles), China (1 article), Switzerland (1 article), and Turkey (1 article). European institutions produced 49 of the most frequently cited articles, North American institutions produced 28, Oceanian institutions produced 21, and Asian institutions produced 2. The Vrije University of Amsterdam and the Curtin University of Australia contributed most frequently to the top 100 cited articles, with 10 and 5 published articles, respectively. Currently, there is only one guideline on palliative care for patients with glioma, which was published in 2017 by the European Association for Neuro-Oncology (EANO) (32).

**Figure 3:**
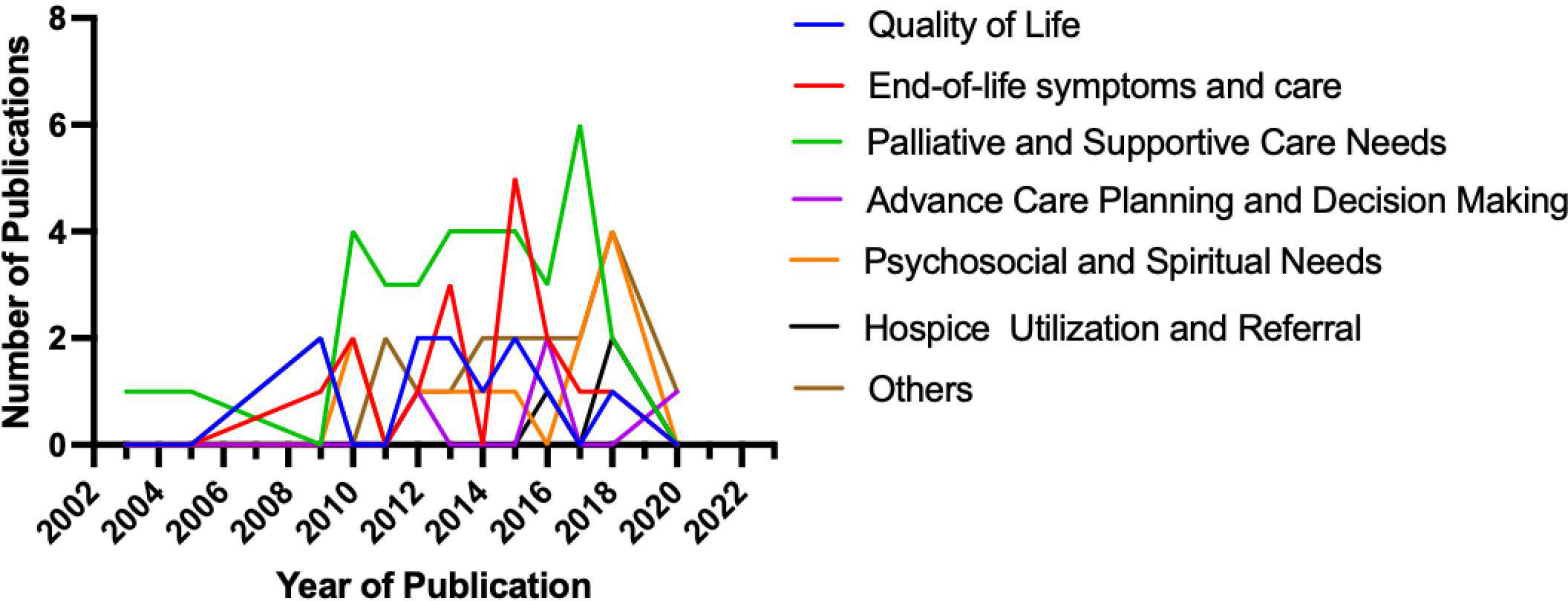
The top 100 cited article categories based on the primary topic.

**Figure 4:**
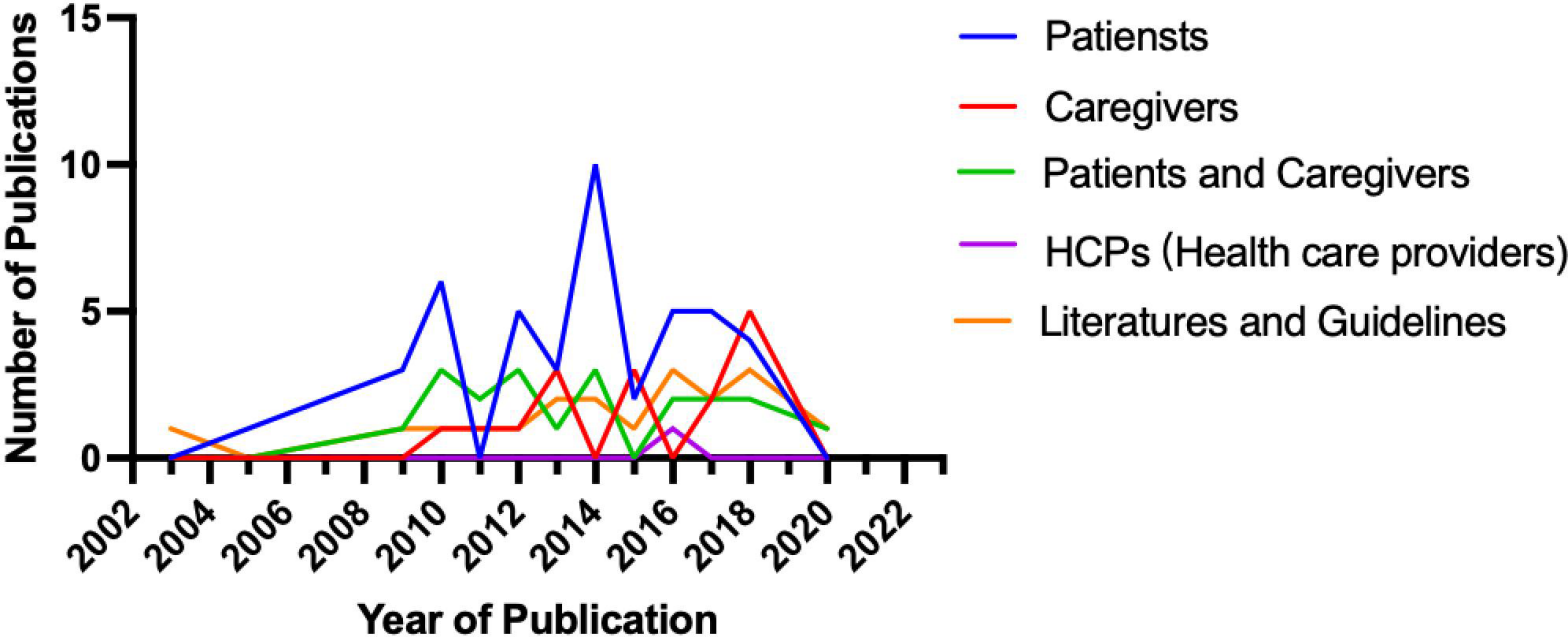
The top 100 cited article categories based on the research subjects.

**Figure 5:**
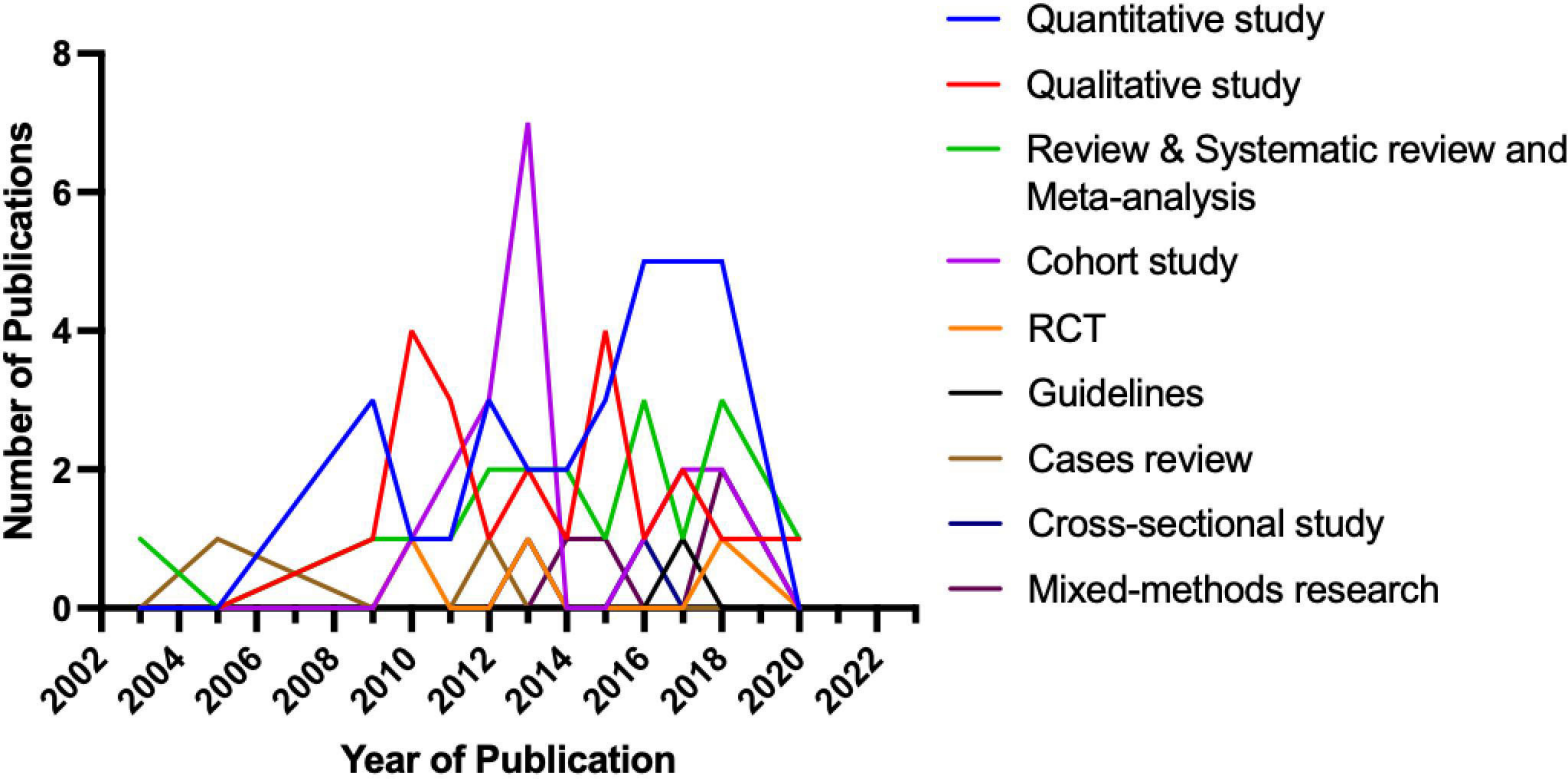
The top 100 cited article categories based on the research methods.

The top 100 cited research articles were subsequently divided into seven major categories based on the primary topic (**Figure 3**): quality of life, end-of-life symptoms and care, palliative and supportive care needs, advance care planning (ACP) and decision-making, psychosocial and spiritual needs, hospice utilization and referral, and others. The studies of the primary topic are correlated with the number of citations (Spearman’s correlation coefficient = −0.206; p < 0.05). End-of-life symptoms and care, patient/caregiver dyads quality of life, and palliative or supportive care needs were the top three most studied topics in the past. Specifically, the top 100 cited articles were divided into the following six major research subject groups: patients, caregivers, patients and caregivers, healthcare providers, the literature, and one palliative care for glioma guideline (**Figure 4**). The studies of the subject are not correlated with the number of citations (Spearman’s correlation coefficient = 0.128; p > 0.05). Furthermore, the top 100 cited articles were divided into the following nine research methods: quantitative study, qualitative study, review or systematic review and meta-analysis, cohort study, RCT, mixed-methods research, cross-sectional study, case review, and one guideline (**Figure 5**).

## Discussion

In this study, we conducted the first scientometric analysis of palliative care issues in patients with glioma. Scientometrics may reflect the degree of attention and research hotspots in a particular field and the quality and impact of the literature. We analyzed the 100 most frequently cited articles and then summarized the basic information. We analyzed the relationship between annual publications and research topics, objects, and methods. We also analyzed research hotspots and alterations in the direction of the field and, to some extent, predicted future research trends in palliative care for gliomas.

We chose the Web of Science database because it not only consists of only institutes for scientific information journals but also has the facility to view and sort articles based on the number of times an article is cited (24). Among the top 100 cited articles, we list the 10 most frequently cited articles in **Table 2**. The core number of times that an article was cited by Web of Science ranged from 71 to 223, and the mean number of citations was 115.4. Between the open-access journals and the standard published journals, there was no statistical difference in the number of citations and the IFs of journals. The IFs of the journals are not correlated with the number of citations. In contrast to glioma treatment, diagnosis, prognosis, and mechanism, the number of citations was fewer, and palliative-specific journals were relatively rare.

**Table 2:** Summary of the top 10 cited articles on palliative for patients with glioma.

Rank	Article title	Citation numbers	Annual citation numbers	Theme
1	Management of glioblastoma: state of the art and future directions	223	111.50	A review: supportive and palliative care are important considerations in the multimodal approach to management
2	Use of video to facilitate end-of-life discussions with patients with cancer: a randomized controlled trial	161	13.42	Goals-of-care video to improve end-of-life decision-making
3	Quality of life in adults with brain tumors: current knowledge and future directions	121	9.31	A review: quality of life and specific symptoms
4	Symptoms and problems in the end-of-life phase of high-grade glioma patients	110	9.17	Symptoms and problems in the end-of-life phase
5	Systematic review of supportive care needs in patients with primary malignant brain tumors	111	11.10	Supportive care needs
6	European Association for Neuro-Oncology (EANO) guidelines for palliative care in adults with glioma	109	21.80	EANO guidelines
7	Prevalence and determinants of depression in caregivers of cancer patients: a systematic review and meta-analysis	91	22.75	Depression and quality of life in caregivers
8	End of life issues in brain tumor patients	85	6.54	Symptoms in the last weeks of disease and EoL decision-making
9	Social, psychological and existential well-being in patients with glioma and their caregivers: a qualitative study	72	7.20	Physical, social, psychological, and existential distress
10	The information and support needs of patients diagnosed with high grade glioma	71	5.92	Information and support needs and patients’ experiences

Europe, North America, and Oceania contributed the most to the top 100 cited articles. Brant JM et al. (33) summarized that some similarities exist in palliative cancer care around the world, but vast differences exist in five primary areas: 1) the epidemiology of cancer and related symptoms experienced, 2) cancer-specific integration into care, 3) palliative care education, 4) economic development of the country, and 5) cultural and religious differences that call for a tailored approach to care. While palliative care services exist in over half of the world’s countries, low-to-middle-income countries (LMICs) are resource-poor and have the fewest resources and least amount of integration of palliative care, especially in remote areas; however, these programs are primarily located in high-income countries (34–36).

The number of citations is a reliable method for quantifying an article’s quality and the impact of its contribution to the scientific community (25). We also analyzed the trend of articles published on primary topics related to palliative care for glioma. Among the top 100 cited articles, **Figure 3** shows the relationship between the publication year of the article and the number of citations on the research themes: patient/caregiver dyads quality of life, end-of-life symptoms and care, palliative and supportive care need, advanced care planning and decision-making, psychosocial and spiritual need, hospice utilization and referral, and others such as caregivers’ economic hardship, patterns of care, social support and resource, sleep characteristics of family caregivers, and anti-epileptic drugs. The studies of the primary topic are correlated with the number of citations, which showed that end-of-life symptoms and care, patient/caregiver dyads quality of life, and palliative or supportive care needs were the top three most studied in the past, whereas the research on end-of-life symptoms and care, and hospice utilization and referral has an increasing trend. In our analysis, the number of studies focusing on patients was higher, while the number of studies focusing on family caregivers or healthcare teams has increased each year. This finding suggests that palliative care is not restricted to patients and encompasses the whole process and all members of the patient’s family and healthcare team. This conclusion indicates that research on palliative care for caregivers and medical teams is a hot topic and a feasible direction for research in the near future.

We further analyzed the publication trend of research subjects. The relationship between the publication year of the article and the number of articles related to research subjects is shown in **Figure 4**. The main research subjects were the patient, caregiver, patient and caregiver, healthcare provider, and literature. The studies of the subject are not correlated with the number of citations. The relationship between the publication year of the article and the number of articles related to research methods is shown in **Figure 5**. Most of the studies performed quantitative analyses, qualitative analyses, systematic analyses, meta-analyses, and retrospective cohort studies. RCTs are less common in palliative care for the glioma field. Numerous clinical studies have documented the benefits of palliative care, and future studies should include additional RCTs to increase the level of evidence.

**Figure 6:**
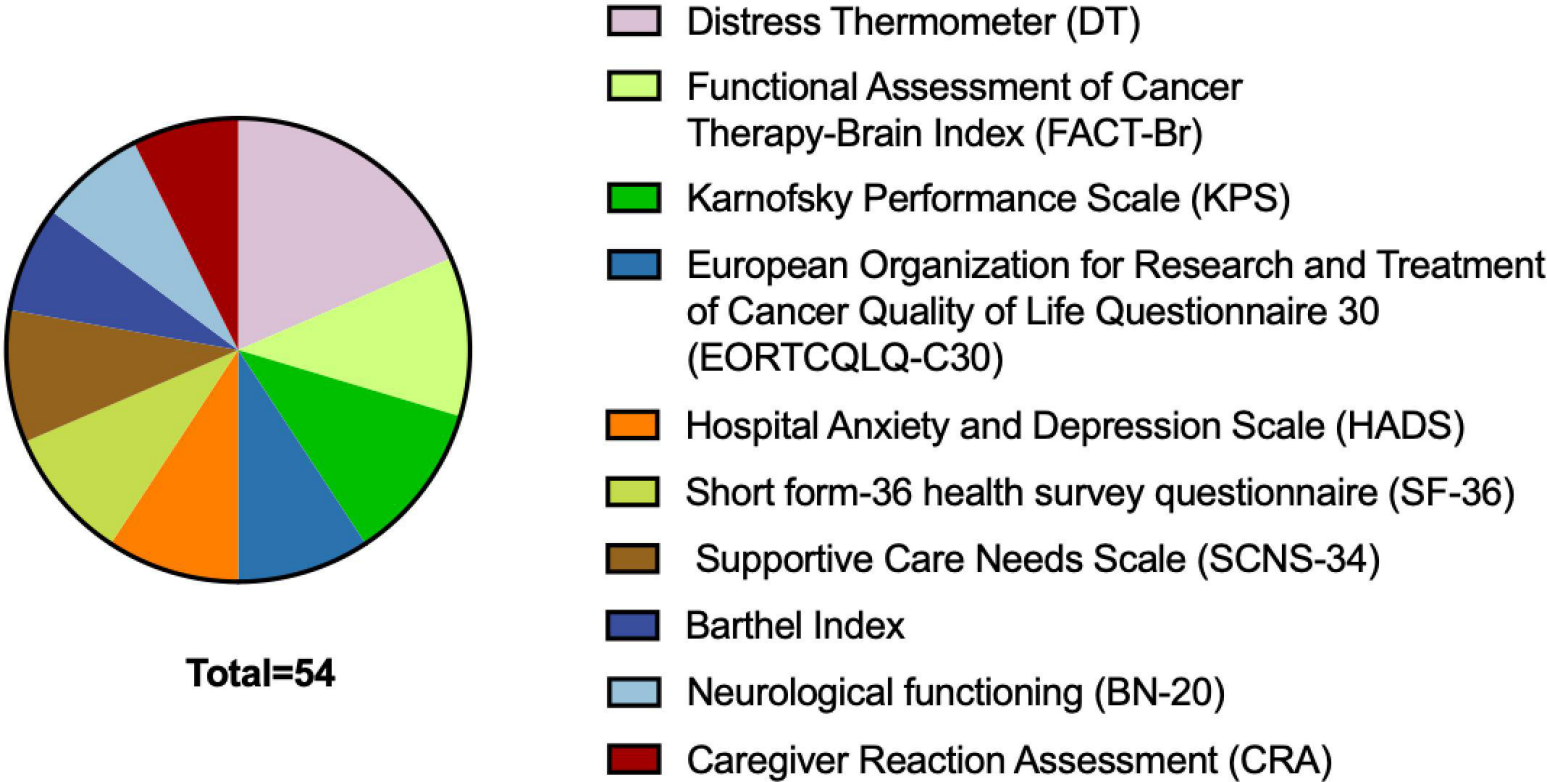
The top 10 scales used in quantitative research.

In quantitative studies, 66 scales were used. The top 10 scales were used 54 times, as shown in **Figure 6**, including the Distress Thermometer (DT), Functional Assessment of Cancer Therapy-Brain Index (FACT-Br), Karnofsky Performance Scale (KPS), European Organization for Research and Treatment of Cancer Quality of Life Questionnaire 30 (EORTCQLQ-C30), Hospital Anxiety and Depression Scale (HADS), Short form-36 health survey questionnaire (SF-36), Supportive Care Needs Scale (SCNS-34), Barthel Index, Neurological functioning (BN-20), and Caregiver Reaction Assessment (CRA). The scales were used in various ways. The same scales were used at different stages of the disease trajectory to observe the trends in the problems being studied. On the one hand, the scales focus on patients’ psychosocial, quality of life, supportive care needs, symptoms and signs during the disease trajectory especially at the end of life (21–23), personality, cognition, and activities of daily living. On the other hand, the scales focus on caregivers’ quality of life, psychological features, multidimensional burden, and support needs. Of the 100 most-cited articles, 12 included both patients and caregivers in the studies. A total of six studies combined scales on patients’ and caregivers’ burden, in which DT, Patient-Generated Index (PGI), and coping strategies (BriefCope) were used twice. MD Anderson Symptom InvenTory-Brain Tumor (MDASI-BT) module and HADS were used one time.

This scientometric analysis of the palliative care for glioma patients 1) helps us grasp the different aspects of palliative care needs of glioma patients and where research should be directed. 2) This is the first scientometric study on palliative care for glioma patients, which makes people realize the importance of palliative care for glioma patients and provides a treatment strategy that can be considered for terminally ill patients. 3) As the spectrum of disease changes and the population ages, palliative care is gradually being recognized and used in different life-limiting conditions; the need for palliative care is increasing, has become an important part of clinical practice, and gradually gained widespread worldwide (37, 38). Palliative care is likely to become a hot topic of research in the future, and the number of journals on this topic will increase.

Our study still has some limitations. First, we used only the Web of Science database for the literature search, which may have resulted in a certain degree of omissions, and annual citation is used to reflect attention which has some errors. Second, the length of the time since publication will affect the citations; some recently published articles were not included due to the low total number of citations. Third, most of the studies used self-report questionnaires and semistructured interviews to observe the problems of the patient and their caregivers, and a retrospective study nature, which may have selection bias and publication bias. In addition, the included population is relatively small, and the population heterogeneity is large, which may affect the results of the study.

## Conclusions

This study provides the first scientometric analysis of palliative care issues of patients with gliomas, enumerates the top 100 most-cited and influential articles, summarizes historical developments, and predicts future research hotspots. We found that the core problem in this field is the palliative care issues of patients with glioma, including research topics and trends, subjects, and research methods. However, the number of RCTs investigating the palliative care of glioma was low, and the evidence is low. Literature reviews and meta-analyses on palliative care for glioma are relatively rare. Future hotspots will mainly focus on RCTs of palliative care for patients with glioma, and a considerable need for high-quality literature reviews and meta-analyses is noted.

## Data availability statement

The original contributions presented in the study are included in the article/**Supplementary material**. Further inquiries can be directed to the corresponding authors.

## Author contributions

Study design and manuscript writing: ZX, WC and HZ. Medical record search and follow-up: HW, BZ, DL, TY, TL and HX. Manuscript formatting and revision: YNW, YKW and XG. All authors contributed to the article and approved the submitted version.
