# Supplementary material for: Palliative care for patients with glioma: A recent scientometric analysis of the Web of Science in 2022
Source: Front Oncol. 2022 Dec 13;12:995639. doi: 10.3389/fonc.2022.995639 (PMC9792968; doi:10.3389/fonc.2022.995639)
Supplement: Supplementary file 1 [file Table_1.docx]

| **Supplementary Table 1**. Summary of the Top 100 Cited Articles on Palliative Care for Glioma | | | | | | | |
| --- | --- | --- | --- | --- | --- | --- | --- |
| **Rank** | **Article Title** | **Authors** | **Years** | **Journal** | **Citation Numbers** | **Article Type** | **Theme** |
| 1 | Management of glioblastoma: State of the art and future directions | Tan, Aaron C.; Ashley, David M.; Lopez, Giselle Y.; et al. | 2020 | CA-A CANCER JOURNAL FOR CLINICIANS | 223 | Review | Management of Glioblastoma |
| 2 | Use of Video to Facilitate End-of-Life Discussions With Patients With Cancer: A Randomized Controlled Trial | El-Jawahri, Areej; Podgurski, Lisa M.; Eichler, April F.; et al. | 2010 | JOURNAL OF CLINICAL ONCOLOGY | 161 | Article | End-of-Life Discussion |
| 3 | Quality of life in adults with brain tumors: Current knowledge and future directions | Liu, Raymond; Page, Margaretta; Solheim, Karla; et al. | 2009 | NEURO-ONCOLOGY | 121 | Review | Quality of Life |
| 4 | Symptoms and problems in the end-of-life phase of high-grade glioma patients | Sizoo, Eefje M.; Braam, Lies; Postma, Tjeerd J.; et al. | 2010 | NEURO-ONCOLOGY | 110 | Article | Symptom and Problem in the End-of-life Phase |
| 5 | Systematic review of supportive care needs in patients with primary malignant brain tumors | Ford, Elizabeth; Catt, Susan; Chalmers, Anthony; et al. | 2012 | NEURO-ONCOLOGY | 111 | Review | Supportive Care Need |
| 6 | European Association for Neuro-Oncology (EANO) guidelines for palliative care in adults with glioma | Pace, Andrea; Dirven, Linda; Koekkoek, Johan A. F.; et al. | 2017 | LANCET ONCOLOGY | 109 | Review | Palliative care Guideline |
| 7 | Prevalence and determinants of depression in caregivers of cancer patients A systematic review and meta-analysis | Geng, Hai-mei; Chuang, Dong-mei; Yang, Fang; et al. | 2018 | MEDICINE | 91 | Review | Depression |
| 8 | End of life issues in brain tumor patients | Pace, Andrea; Di Lorenzo, Cherubino; Guariglia, Lara; et al. | 2009 | JOURNAL OF NEURO-ONCOLOGY | 85 | Article | End of life Issue |
| 9 | Social, psychological and existential well-being in patients with glioma and their caregivers: a qualitative study | Cavers, Debbie; Hacking, Belinda; Erridge, Sara E.; et al. | 2012 | CANADIAN MEDICAL ASSOCIATION JOURNAL | 72 | Article | Social, Psychological and Existential Well-being |
| 10 | The information and support needs of patients diagnosed with High Grade Glioma | Halkett, Georgia K. B.; Lobb, Elizabeth A.; Oldham, Lynn; et al. | 2010 | PATIENT EDUCATION AND COUNSELING | 71 | Article | Information and Support need |
| 11 | The impact of a high-grade glioma on everyday life: A systematic review from the patient's and caregiver's perspective | Sterckx, Wendy; Coolbrandt, Annemarie; de Casterle, Bernadette Dierckx; et al. | 2013 | EUROPEAN JOURNAL OF ONCOLOGY NURSING | 71 | Review | Information and support need |
| 12 | Caring for someone with high-grade glioma: a time of rapid change for caregivers | McConigley, Ruth; Halkett, Georgia; Lobb, Elizabeth; et al. | 2010 | PALLIATIVE MEDICINE | 69 | Article | Information and Support Need |
| 13 | Communication, information and support for adults with malignant cerebral glioma: a systematic literature review | Davies, E; Higginson, IJ | 2003 | SUPPORTIVE CARE IN CANCER | 68 | Review | Communication, Information and Support |
| 14 | Decision-making in the end-of-life phase of high-grade glioma patients | Sizoo, Eefje M.; Pasman, H. Roeline W.; Buttolo, Janine; et al. | 2012 | EUROPEAN JOURNAL OF CANCER | 62 | Article | Decision-making |
| 15 | Enhancing quality of life and mastery of informal caregivers of high-grade glioma patients: a randomized controlled trial | Boele, Florien W.; Hoeben, Wopke; Hilverda, Karen; et al. | 2013 | JOURNAL OF NEURO-ONCOLOGY | 57 | Article | Quality of Life |
| 16 | Palliative care of patients with a primary malignant brain tumour: case review of service use and support provided | Faithfull, S; Cook, K; Lucas, C | 2005 | PALLIATIVE MEDICINE | 61 | Review | Palliative care utilized |
| 17 | Identifying the palliative care needs of patients living with cerebral tumors and metastases: a retrospective analysis | Gofton, Teneille E.; Graber, Jerome; Carver, Alan | 2012 | JOURNAL OF NEURO-ONCOLOGY | 57 | Article | Symptom and End of Life Care Decision, Palliative Care Need |
| 18 | Differential palliative care issues in patients with primary and secondary brain tumours | Ostgathe, Christoph; Gaertner, Jan; Kotterba, Maren; et al. | 2010 | SUPPORTIVE CARE IN CANCER | 55 | Article | Symptom and Palliative Care Need |
| 19 | End-of-life symptoms and care in patients with primary malignant brain tumors: a systematic literature review | Walbert, Tobias; Khan, Muhib | 2014 | JOURNAL OF NEURO-ONCOLOGY | 55 | Review | End-of-life Symptoms and Care |
| 20 | The symptom burden of primary brain tumors: evidence for a core set of tumor- and treatment-related symptoms | Armstrong, Terri S.; Vera-Bolanos, Elizabeth; Acquaye, Alvina A.; et al. | 2016 | NEURO-ONCOLOGY | 54 | Article | Symptom Burden |
| 21 | Patient and caregiver perceptions of communication of prognosis in high grade glioma | Lobb, E. A.; Halkett, G. K. B.; Nowak, A. K. | 2011 | JOURNAL OF NEURO-ONCOLOGY | 51 | Article | Prognostic Information |
| 22 | The spiritual needs of neuro-oncology patients from patients' perspective | Nixon, Aline; Narayanasamy, Aru | 2010 | JOURNAL OF CLINICAL NURSING | 55 | Article | Spiritual Need |
| 23 | Distress and quality of life in primary high-grade brain tumor patients | Kvale, Elizabeth A.; Murthy, Rashmi; Taylor, Richard; et al. | 2009 | SUPPORTIVE CARE IN CANCER | 51 | Article | Distress and Quality of life |
| 24 | The end-of-life phase of high-grade glioma patients: a systematic review | Sizoo, Eefje M.; Pasman, H. Roeline W.; Dirven, Linda; et al. | 2014 | SUPPORTIVE CARE IN CANCER | 46 | Review | End-of-life phase Issues (Symptoms, Supportive Medication, Advance Care Planning and Decision-Making) |
| 25 | Quality of Care and Rehospitalization Rate in the Last Stage of Disease in Brain Tumor Patients Assisted at Home: A Cost Effectiveness Study | Pace, Andrea; Di Lorenzo, Cherubino; Capon, Alessandra; et al. | 2012 | JOURNAL OF PALLIATIVE MEDICINE | 46 | Article | Quality of Care and Rehospitalization Rate |
| 26 | The caregivers' perspective on the end-of-life phase of glioblastoma patients | Flechl, Birgit; Ackerl, Michael; Sax, Cornelia; et al. | 2013 | JOURNAL OF NEURO-ONCOLOGY | 44 | Article | End-of-life Phase Issue |
| 27 | Palliative and supportive care needs of patients with high-grade glioma and their carers: A systematic review of qualitative literature | Moore, Gaye; Collins, Anna; Brand, Caroline; et al. | 2013 | PATIENT EDUCATION AND COUNSELING | 46 | Review | Palliative and Supportive Care Need |
| 28 | The End-of-Life Phase of High-Grade Glioma Patients: Dying With Dignity? | Sizoo, Eefje M.; Taphoorn, Martin J. B.; Uitdehaag, Bernard; et al. | 2013 | ONCOLOGIST | 44 | Article | End-of-life Phase Issues |
| 29 | Needs for everyday life support for brain tumour patients' relatives: systematic literature review | Madsen, K.; Poulsen, H. S. | 2011 | EUROPEAN JOURNAL OF CANCER CARE | 44 | Review | Supportive Care Need |
| 30 | Rates and risks for late referral to hospice in patients with primary malignant brain tumors | Diamond, Eli L.; Russell, David; Kryza-Lacombe, Maria; et al. | 2016 | NEURO-ONCOLOGY | 42 | Article | Hospice Referral |
| 31 | Living with a brain tumor Reaction profiles in patients and their caregivers | Petruzzi, Alessandra; Finocchiaro, Claudia Yvonne; Lamperti, Elena; et al. | 2013 | SUPPORTIVE CARE IN CANCER | 39 | Article | Quality of Life |
| 32 | Predictors of distress and poorer quality of life in High Grade Glioma patients | Halkett, Georgia K. B.; Lobb, Elizabeth A.; Rogers, Michelle M.; et al. | 2015 | PATIENT EDUCATION AND COUNSELING | 36 | Article | Quality of Life, Distress |
| 33 | Health-related quality of life in high-grade glioma patients: a prospective single-center study | Yavas, Cagdas; Zorlu, Faruk; Ozyigit, Gokhan; et al. | 2012 | SUPPORTIVE CARE IN CANCER | 38 | Article | Quality of Life |
| 34 | Primary brain tumor patients' supportive care needs and multidisciplinary rehabilitation, community and psychosocial support services: awareness, referral and utilization | Langbecker, Danette; Yates, Patsy | 2016 | JOURNAL OF NEURO-ONCOLOGY | 35 | Article | Supportive Care Need  Community and Psychosocial Support Service |
| 35 | Controlled rehabilitative and supportive care intervention trials in patients with high-grade gliomas and their caregivers: a systematic review | Piil, K.; Juhler, M.; Jakobsen, J.; et al. | 2016 | BMJ SUPPORTIVE & PALLIATIVE CARE | 34 | Review | Supportive Care |
| 36 | Mapping the patterns of care, the receipt of palliative care and the site of death for patients with malignant glioma | Sundararajan, Vijaya; Bohensky, Megan A.; Moore, Gaye; et al. | 2014 | JOURNAL OF NEURO-ONCOLOGY | 34 | Article | Palliative Care and The Site of Death, Patterns of Care |
| 37 | Family Caregivers' Level of Mastery Predicts Survival of Patients With Glioblastoma: A Preliminary Report | Boele, Florien W.; Given, Charles W.; Given, Barbara A.; et al. | 2017 | CANCER | 33 | Article | Caregivers'  Psychological and Mastery |
| 38 | Adjustment and support needs of glioma patients and their relatives: serial interviews | Cavers, Debbie; Hacking, Belinda; Erridge, Sara C.; et al. | 2013 | PSYCHO-ONCOLOGY | 33 | Article | Support, Information Need |
| 39 | Changes in Caregiver Perceptions Over Time in Response to Providing Care for a Loved One With a Primary Malignant Brain Tumor | Sherwood, Paula; Hricik, Allison; Donovan, Heidi; et al. | 2011 | ONCOLOGY NURSING FORUM | 33 | Article | Caregiver Need |
| 40 | Making sense of brain tumour: A qualitative investigation of personal and social processes of adjustment | Ownsworth, Tamara; Chambers, Suzanne; Hawkes, Anna; et al. | 2011 | NEUROPSYCHOLOGICAL REHABILITATION | 33 | Article | Personal and Social Process |
| 41 | Life beyond a diagnosis of glioblastoma: a systematic review of the literature | Gately, L.; McLachlan, S. A.; Dowling, A.; Philip, J. | 2017 | JOURNAL OF CANCER SURVIVORSHIP | 32 | Review | Care Needs and Support Network |
| 42 | Screening for psychological distress in adult primary brain tumor patients and caregivers: considerations for cancer care coordination | Trad, Wafa; Koh, Eng-Siew; Daher, Maysaa; et al. | 2015 | FRONTIERS IN ONCOLOGY | 31 | Article | Psychological Distress |
| 43 | I'm just waiting...: an exploration of the experience of living and dying with primary malignant glioma | Philip, Jennifer; Collins, Anna; Brand, Caroline A.; et al. | 2014 | SUPPORTIVE CARE IN CANCER | 31 | Article | Supportive and Palliative Care Need |
| 44 | Supportive care in neurooncology | Pace, Andrea; Metro, Giulio; Fabi, Alessandra | 2010 | CURRENT OPINION IN ONCOLOGY | 32 | Review | Supportive Care |
| 45 | Sleep Characteristics of Family Caregivers of Individuals With a Primary Malignant Brain Tumor | Pawl, Jean D.; Lee, Shih-Yu; Clark, Patricia C.; et al. | 2013 | ONCOLOGY NURSING FORUM | 30 | Article | Sleep Characteristics of Family Caregivers |
| 46 | Distress and psychological morbidity do not reduce over time in carers of patients with high-grade glioma | Halkett, Georgia K. B.; Lobb, Elizabeth A.; Shaw, Therese; et al. | 2017 | SUPPORTIVE CARE IN CANCER | 27 | Article | Distress and Psychological |
| 47 | The multidimensional burden of informal caregivers in primary malignant brain tumor | Bayen, Eleonore; Laigle-Donadey, Florence; Proute, Myrtille; et al. | 2017 | SUPPORTIVE CARE IN CANCER | 27 | Article | ICs Burdens |
| 48 | Advance Care Planning in Patients with Primary Malignant Brain Tumors: A Systematic Review | Song, Krystal; Amatya, Bhasker; Voutier, Catherine; et al. | 2016 | FRONTIERS IN ONCOLOGY | 28 | Review | Advance Care Planning |
| 49 | The burden of brain tumor: a single-institution study on psychological patterns in caregivers | Finocchiaro, Claudia Yvonne; Petruzzi, Alessandra; Lamperti, Elena; et al. Gaviani, Paola; Silvani, Antonio; Sarno, Lucio; et al. | 2012 | JOURNAL OF NEURO-ONCOLOGY | 27 | Article | Caregiver’s Quality of Life and Psychosocial Well-being |
| 50 | Identifying the needs of brain tumor patients and their caregivers | Parvataneni, Rupa; Polley, Mei-Yin; Freeman, Teresa; et al. | 2011 | JOURNAL OF NEURO-ONCOLOGY | 27 | Article | Supportive Needs and Psychosocial Support |
| 51 | Perceptions of economic hardship and emotional health in a pilot sample of family caregivers | Bradley, Sarah E.; Sherwood, Paula R.; Kuo, Jean; et al. | 2009 | JOURNAL OF NEURO-ONCOLOGY | 27 | Article | Caregivers Economic Hardship and Emotional Health |
| 52 | Daily Life Experiences of Patients With a High-Grade Glioma and Their Caregivers: A Longitudinal Exploration of Rehabilitation and Supportive Care Needs | Piil, Karin; Juhler, Marianne; Jakobsen, Johannes; et al. | 2015 | JOURNAL OF NEUROSCIENCE NURSING | 26 | Article | Rehabilitation and Supportive Care Need |
| 53 | Epilepsy in the end-of-life phase in patients with high-grade gliomas | Pace, Andrea; Villani, Veronica; Di Lorenzo, Cherubino; et al. | 2013 | JOURNAL OF NEURO-ONCOLOGY | 27 | Article | Epilepsy |
| 54 | Needs and preferences among patients with high-grade glioma and their caregivers - A longitudinal mixed methods study | Piil, K.; Jakobsen, J.; Christensen, K. B.; et al. | 2018 | EUROPEAN JOURNAL OF CANCER CARE | 25 | Article | Need and Preference |
| 55 | Impact of provider level, training and gender on the utilization of palliative care and hospice in neuro-oncology: a North-American survey | Walbert, Tobias; Glantz, Michael; Schultz, Lonni; et al. | 2016 | JOURNAL OF NEURO-ONCOLOGY | 25 | Article | The Utilization of Palliative Care and Hospice |
| 56 | Health-related quality of life in patients with high-grade gliomas: a quantitative longitudinal study | Piil, K.; Jakobsen, J.; Christensen, K. B.; et al. | 2015 | JOURNAL OF NEURO-ONCOLOGY | 25 | Article | Health-related Quality of Life |
| 57 | Living with a high-grade glioma: A qualitative study of patients' experiences and care needs | Sterckx, Wendy; Coolbrandt, Annemarie; Clement, Paul; et al. | 2015 | EUROPEAN JOURNAL OF ONCOLOGY NURSING | 25 | Article | Patients' Experience and Care Need |
| 58 | Hospitalization burden and survival among older glioblastoma patients | Arvold, Nils D.; Wang, Yun; Zigler, Cory; et al. | 2014 | NEURO-ONCOLOGY | 24 | Article | Hospitalization Burden |
| 59 | Symptoms and medication management in the end of life phase of high-grade glioma patients | Koekkoek, J. A. F.; Dirven, L.; Sizoo, E. M.; et al. | 2014 | JOURNAL OF NEURO-ONCOLOGY | 24 | Article | Symptoms and Medication Management in the End of Life Phase |
| 60 | Finding the right kind of support: A study of carers of those with a primary malignant brain tumour | Arber, Anne; Hutson, Nicky; de Vries, Kay; et al. | 2013 | EUROPEAN JOURNAL OF ONCOLOGY NURSING | 24 | Article | Support Need of Carers |
| 61 | Odyssey of hope: a physician's guide to communicating with brain tumor patients across the continuum of care | Rosenblum, Mark L.; Kalkanis, Steven; Goldberg, Wendy; et al. | 2009 | JOURNAL OF NEURO-ONCOLOGY | 24 | Editorial Material | Physician's Guide to Communicating |
| 62 | Advance Care Planning in Glioblastoma Patients | Fritz, Lara; Dirven, Linda; Reijneveld, Jaap C.; et al. | 2016 | CANCERS | 22 | Review | Advance Care Planning |
| 63 | Evaluating patients for psychosocial distress and supportive care needs based on health-related quality of life in primary brain tumors: a prospective multicenter analysis of patients with gliomas in an outpatient setting | Hickmann, Anne-Katrin; Hechtner, Marlene; Nadji-Ohl, Minou; et al. | 2017 | JOURNAL OF NEURO-ONCOLOGY | 21 | Article | Psychosocial Distress and Supportive Care Need |
| 64 | Attitudes and preferences toward monitoring symptoms, distress, and quality of life in glioma patients and their informal caregivers | Boele, Florien W.; van Uden-Kraan, Cornelia F.; Hilverda, Karen; et al. | 2016 | SUPPORTIVE CARE IN CANCER | 22 | Article | Quality of Life |
| 65 | Support after brain tumor means different things: family caregivers' experiences of support and relationship changes | Ownsworth, Tamara; Goadby, Elizabeth; Chambers, Suzanne Kathleen | 2015 | FRONTIERS IN ONCOLOGY | 21 | Article | Caregivers' Experience of Support and Relationship Changes |
| 66 | Home palliative care and end of life issues in glioblastoma multiforme: results and comments from a homogeneous cohort of patients | Pompili, Alfredo; Telera, Stefano; Villani, Veronica; et al. | 2014 | NEUROSURGICAL FOCUS | 21 | Article | Home palliative care and End of Life Issues |
| 67 | End-of-life caregivers perception of medical and psychological support during the final weeks of glioma patients: a questionnaire-based survey | Heese, Oliver; Vogeler, Eva; Martens, Tobias; et al. | 2013 | NEURO-ONCOLOGY | 23 | Article | End-of-life Caregivers Perception of Medical and Psychological Support |
| 68 | Neuro-oncology and palliative care: a challenging interface | Lin, Esther; Rosenthal, Mark A.; Le, Brian H.; et al. | 2012 | NEURO-ONCOLOGY | 21 | Article | Palliative Care |
| 69 | Psychosocial Implications for the Patient With a High-Grade Glioma | Lucas, Michele R. | 2010 | JOURNAL OF NEUROSCIENCE NURSING | 22 | Article | Psychosocial |
| 70 | End-of-Life Care in High-Grade Glioma Patients. The Palliative and Supportive Perspective | Giammalva, Giuseppe Roberto; Iacopino, Domenico Gerardo; Azzarello, Giorgio; et al. | 2018 | BRAIN SCIENCES | 21 | Review | End-of-Life Care Palliative and Supportive Perspective |
| 71 | Do carer's levels of unmet needs change over time when caring for patients diagnosed with high-grade glioma and how are these needs correlated with distress? | Halkett, G. K. B.; Lobb, E. A.; Shaw, T.; et al. | 2018 | SUPPORTIVE CARE IN CANCER | 20 | Article | Carer's Need and Distress |
| 72 | Assessing psychological and supportive care needs in glioma patients - feasibility study on the use of the Supportive Care Needs Survey Short Form (SCNS-SF34-G) and the Supportive Care Needs Survey Screening Tool (SCNS-ST9) in clinical practice | Renovanz, M.; Hickmann, A. -K.; Coburger, J.; et al. | 2018 | EUROPEAN JOURNAL OF CANCER CARE | 20 | Article | Psychological and Supportive Care Need |
| 73 | Factors associated with supportive care needs in glioma patients in the neuro-oncological outpatient setting | Renovanz, Mirjam; Hechtner, Marlene; Janko, Mareile; et al. | 2017 | JOURNAL OF NEURO-ONCOLOGY | 20 | Article | Supportive Care Need |
| 74 | Antiepileptic drug treatment in the end-of-life phase of glioma patients: a feasibility study | Koekkoek, Johan A. F.; Postma, Tjeerd J.; Heimans, Jan J.; et al. | 2016 | SUPPORTIVE CARE IN CANCER | 20 | Article | Antiepileptic Drug |
| 75 | End of life care in high-grade glioma patients in three European countries: a comparative study | Koekkoek, J. A. F.; Dirven, L.; Reijneveld, J. C.; et al. | 2014 | JOURNAL OF NEURO-ONCOLOGY | 20 | Article | End-of-life Care |
| 76 | Hospice utilization in patients with malignant gliomas | Forst, Deborah; Adams, Eric; Nipp, Ryan; et al. | 2018 | NEURO-ONCOLOGY | 19 | Article | Hospice Utilization |
| 77 | Prognostic awareness and communication preferences among caregivers of patients with malignant glioma | Applebaum, A. J.; Buda, K.; Kryza-Lacombe, M.; et al. | 2018 | PSYCHO-ONCOLOGY | 19 | Article | Prognostic Awareness and Communication Preference |
| 78 | The Last 10 Days of Patients With Glioblastoma: Assessment of Clinical Signs and Symptoms as well as Treatment | Thier, Katrin; Calabek, Bernadette; Tinchon, Alexander; et al. | 2016 | AMERICAN JOURNAL OF HOSPICE & PALLIATIVE MEDICINE | 19 | Article | Last 10 Days of Clinical Signs and Symptoms and Treatment |
| 79 | Exploring the Support Needs of Family Caregivers of Patients with Brain Cancer Using the CSNAT: A Comparative Study with Other Cancer Groups | Aoun, Samar M.; Deas, Kathleen; Howting, Denise; et al. | 2015 | PLOS ONE | 21 | Article | Support Need |
| 80 | Systematic review of interventions to improve the provision of information for adults with primary brain tumors and their caregivers | Langbecker, Danette; Janda, Monika | 2015 | FRONTIERS IN ONCOLOGY | 18 | Review | Information Provision |
| 81 | Coping strategies and quality of life: a longitudinal study of high-grade glioma patient-caregiver dyads | Baumstarck, Karine; Chinot, Olivier; Tabouret, Emeline; et al. | 2018 | HEALTH AND QUALITY OF LIFE OUTCOMES | 18 | Article | Coping Strategy and Quality of life |
| 82 | A proposed framework of supportive and palliative care for people with high-grade glioma | Philip, Jennifer; Collins, Anna; Brand, Caroline; et al. | 2018 | NEURO-ONCOLOGY | 17 | Article | Supportive and Palliative care |
| 83 | Don't need help, don't want help, can't get help: How patients with brain tumors account for not using rehabilitation, psychosocial and community services | Langbecker, Danette; Ekberg, Stuart; Yates, Patsy | 2017 | PATIENT EDUCATION AND COUNSELING | 17 | Article | Rehabilitation, Psychosocial and Community Service |
| 84 | Coping with a newly diagnosed high-grade glioma: patient-caregiver dyad effects on quality of life | Baumstarck, K.; Leroy, T.; Hamidou, Z.; et al. | 2016 | JOURNAL OF NEURO-ONCOLOGY | 16 | Article | Quality of Life |
| 85 | Congruence of Primary Brain Tumor Patient and Caregiver Symptom Report | Armstrong, Terri S.; Wefel, Jeffrey S.; Gning, Ibrahima; et al. | 2012 | CANCER | 16 | Article; Proceedings Paper | Patient and Caregiver Symptom Report |
| 86 | The views of patients with brain cancer about palliative care: a qualitative study | Vierhout, M.; Daniels, M.; Mazzotta, P.; et al. | 2017 | CURRENT ONCOLOGY | 15 | Article | Palliative Care |
| 87 | Measuring health-related quality of life in high-grade glioma patients at the end of life using a proxy-reported retrospective questionnaire | Sizoo, Eefje M.; Dirven, Linda; Reijneveld, Jaap C.; et al. | 2014 | JOURNAL OF NEURO-ONCOLOGY | 15 | Article | Health-related Quality of Life |
| 88 | Family Caregivers of Patients With a High-Grade Glioma A Qualitative Study of Their Lived Experience and Needs Related to Professional Care | Coolbrandt, Annemarie; Sterckx, Wendy; Clement, Paul; et al. | 2015 | CANCER NURSING | 15 | Article | Caregivers Lived Experience and Needs |
| 89 | Glioblastoma multiforme from diagnosis to death: a prospective, hospital-based, cohort, pilot feasibility study of patient reported symptoms and needs | Golla, Heidrun; Ahmad, Maryam Ale; Galushko, Maren; et al. | 2014 | SUPPORTIVE CARE IN CANCER | 14 | Article | Symptoms and Needs |
| 90 | End of life care for glioblastoma patients at a large academic cancer center | Kuchinad, Kamini E.; Strowd, Roy; Evans, Anne; et al. | 2017 | JOURNAL OF NEURO-ONCOLOGY | 13 | Article | End-of-life Care |
| 91 | Clinical presentation and patterns of care for short-term survivors of malignant glioma | Collins, Anna; Sundararajan, Vijaya; Brand, Caroline A.; et al. | 2014 | JOURNAL OF NEURO-ONCOLOGY | 13 | Article | Palliative and Supportive Care |
| 92 | Mediating burden and stress over time: Caregivers of patients with primary brain tumor | Reblin, Maija; Small, Brent; Jim, Heather; et al. | 2018 | PSYCHO-ONCOLOGY | 12 | Article | Ameliorating the Burden and Stress |
| 93 | The social trajectory of brain tumor: a qualitative metasynthesis | Cubis, Lee; Ownsworth, Tamara; Pinkham, Mark B.; et al. | 2018 | DISABILITY AND REHABILITATION | 12 | Review | Social Network |
| 94 | Patterns of care at end of life for people with primary intracranial tumors: lessons learned | Alturki, Abdulrahman; Gagnon, Bruno; Petrecca, Kevin; et al. | 2014 | JOURNAL OF NEURO-ONCOLOGY | 12 | Article | Patterns of Care |
| 95 | Together and apart: providing psychosocial support for patients and families living with brain tumors | Kanter, Cheryl; D'Agostino, Norma Mammone; Daniels, Maureen; et al. | 2014 | SUPPORTIVE CARE IN CANCER | 12 | Article | Psychosocial Support |
| 96 | Advance care planning in glioblastoma patients: development of a disease-specific ACP program | Fritz, Lara; Zwinkels, Hanneke; Koekkoek, Johan A. F.; et al. | 2020 | SUPPORTIVE CARE IN CANCER | 11 | Article | Advance Care Planning |
| 97 | Feasibility of implementing an electronic social support and resource visualization tool for caregivers in a neuro-oncology clinic | Reblin, Maija; Ketcher, Dana; Forsyth, Peter; et al. | 2018 | SUPPORTIVE CARE IN CANCER | 11 | Article | Social Support and Resource |
| 98 | High psychosocial burden in relatives of malignant brain tumor patients | Stieb, Sonja; Fischbeck, Sabine; Wagner, Wolfgang; et al. | 2018 | CLINICAL NEUROLOGY AND NEUROSURGERY | 11 | Article | Psychosocial |
| 99 | Screening for symptom burden and supportive needs of patients with glioblastoma and brain metastases and their caregivers in relation to their use of specialized palliative care | Seekatz, Bettina; Lukasczik, Matthias; Loehr, Mario; Ehrmann, Katja; et al. | 2017 | SUPPORTIVE CARE IN CANCER | 11 | Article | Symptom Burden and Supportive Need |
| 100 | Hospice care, cancer-directed therapy, and Medicare expenditures among older patients dying with malignant brain tumors | Dover, Laura L.; Dulaney, Caleb R.; Williams, Courtney P.; et al. | 2018 | NEURO-ONCOLOGY | 10 | Article | End-of-life Utilization of Hospice Care |
